# Human Physiology During Exposure to the Cave Environment: A Systematic Review With Implications for Aerospace Medicine

**DOI:** 10.3389/fphys.2019.00442

**Published:** 2019-04-24

**Authors:** Lucrezia Zuccarelli, Letizia Galasso, Rachel Turner, Emily J. B. Coffey, Loredana Bessone, Giacomo Strapazzon

**Affiliations:** ^1^Department of Medicine, University of Udine, Udine, Italy; ^2^Directorate of Human and Robotics, Exploration, European Space Agency, Köln, Germany; ^3^Department of Biomedical Science for Health, University of Milan, Milan, Italy; ^4^Institute of Mountain Emergency Medicine, Eurac Research, Bolzano, Italy; ^5^Department of Psychology, Concordia University, Montreal, QC, Canada

**Keywords:** space analog, spaceflight, extreme environments, circadian rhythms, human physiology, cave

## Abstract

**Background:** Successful long-duration missions outside low-Earth orbit will depend on technical and physiological challenges under abnormal environmental conditions. Caves, characterized by absence of light, confinement, three-dimensional human movement and long-duration isolation, are identifiably one of the earliest examples of scientific enquiry into space analogs. However, little is known about the holistic human physiological response during cave exploration or prolonged habitation.

**Objectives:** The aim of our review was to conduct a systematic bibliographic research review of the effects of short and prolonged exposure to a cave environment on human physiology, with a view to extend the results to implications for human planetary exploration missions.

**Methods:** A systematic search was conducted following the structured PRISMA (Preferred Reporting Items for Systematic Reviews and Meta-Analyses) guidelines for electronic databases.

**Results:** The search retrieved 1,519 studies. There were 50 articles selected for further consideration, of which 31 met our inclusion criteria. Short-term cave exposure studies have investigated visual dysfunction, cardiovascular, endocrine-metabolic, immunologic-hematological and muscular responses in humans. Augmentations of heart rate, muscular damage, initial anticipatory stress reaction and inflammatory responses were reported during caving activity. Prolonged exposure studies mainly investigated whether biological rhythms persist or desist in the absence of standard environmental conditions. Changes were evident in estimated vs. actual rest-activity cycle periods and external desynchronization, body temperature, performance reaction time and heart rate cycles. All studies have shown a marked methodological heterogeneity and lack reproduction under controlled conditions.

**Conclusions:** This review facilitates a further comparison of the proposed physiological impact of a subterranean space analog environment, with existing knowledge in related disciplines pertaining to human operative preparation under challenging environmental conditions. This comprehensive overview should stimulate more reproducible research on this topic and offer the opportunity to advance study design and focus future human research in the cave environment on noteworthy, reproducible projects.

## Introduction

### Rationale

Long duration exploration and future human and robotic planetary surface missions are one of the long-term goals of space agencies (Robinson et al., 2007; Foing, 2016). Preparing for exploration-class missions to other planets requires a close replication of potential environmental and situational characteristics of extreme space conditions. Terrestrial space analogs offer a controlled environment for space research without leaving the planet (Pagel and Choukér, 2016). Since many years, isolation and confinement studies have been conducted in simulated scenarios such as the Mars500, Envihab and PlanHab facilities (Koch and Gerzer, 2008; Meigal and Fomina, 2016; Rittweger et al., 2016; Salvadego et al., 2018). In addition, real world scenarios based in extreme environmental conditions have been adapted to closely replicate the challenges of space exploration. What was previously the territory of only bold, enthusiastic explorers or honed ultra-endurance athletes has become the permanent training environment for the next generation of human elite space pioneers and clinicians. Simulated expeditions have been established in space analog locations around the globe, such as NASA Extreme Environment Mission Operations (NEEMO), Cooperative Adventure for Valuing and Exercising human behavior and performance Skills (ESA CAVES) and Antarctic programs in existing research stations (i.e., Concordia) (Lugg, 2005; Bessone et al., 2013; Anglin and Kring, 2016).

As a priority, better definition and investigation of physiological and psychosomatic effects of long-term permanence in space on the human body is required, as human performance directly impacts on safety and the mission outcome. In this regard, isolation and confinement specific to space habitat simulations have previously been identified as factors that heavily impact on the homeostatic regulation of human physiology, inclusive of various biological variables (Czeisler and Gooley, 2007), as well as behavioral health, cognitive and physical performance (Basner et al., 2014; Pagel and Choukér, 2016; Mogilever et al., 2018). Specifically, alteration of the biological rhythm (Pittendrigh, 1960) was one of the first topics, and is still of great interest to space agencies worldwide (Czeisler and Gooley, 2007; Robinson et al., 2007; Foing, 2016). Caves are one of the earliest scenarios where investigations specifying long-duration confined-isolation were conducted. As early as 1938, well before the launch of any Antarctic winter-over observational studies, Kleitman and his assistant spent 32 days in the Mammoth Cave, Kentucky, US, to specifically investigate how the absence of natural environmental time cues affects the circadian rhythm (Kleitman, 1963). Later isolation studies by Siffre and Montalbini attracted the interest of space agencies in the late 1970s (Siffre, 1988, 1990; Sonnenfeld et al., 1992). Despite this early interest and the inclusion of caves as an analog environment into the ESA astronaut training program since 2011 (Strapazzon et al., 2014; Mogilever et al., 2018), little data have been published in peer-reviewed journals since, and no systematic review exists of the work completed to date from a physiological or clinical perspective. International space agencies worldwide, motivated by a vision for long-term extra-terrestrial habitation and exploration, have expressed a need to better understand the potential effects of this complex cave space analog on the physiological functionality of trained astronauts. An analog which allows the direct investigation of individuals specifically trained to concurrently perform complex physical tasks and fulfill explorative responsibilities, while exposed to an enclosed, dark, atmospherically challenging, hot and humid environment.

### Objectives

The aim of this work is to systematically review studies investigating the effects of short and prolonged exposure to a cave environment on human physiology, to facilitate the greater understanding of all available primary research in this field and extend the results to the implications for human planetary exploration missions and space medicine considerations in the future.

## Methods

### Identification and Protocol

A systematic review of the literature was conducted following the PRISMA (Preferred Reporting Items for Systematic Reviews and Meta-Analyses) Guidelines for Systematic Review (Liberati et al., 2009).

### Eligibility Criteria

An extensive literature search was performed using recognized life science and biomedical electronic databases and by manually searching reference lists of those articles found, which specifically investigate human physiological responses to the cave environment. No language, publication date, or publication status restrictions were imposed. This search was applied to the following electronic databases: Medline (1966-Present), NASA Technical Reports Server (1915-Present), Google Scholar (all electronic resources-Present), Worldcat (1971-Present), OPAC (1831-Present), and PubMed (1997- Present). The last search was run on 24 November 2017.

### Information Sources and Search Strategy

Three members of the project team developed the search strategy (GS, LB, LZ). The search concepts included studies in the cave environment; the following search approach was used: 01. “cave/s” [All Fields], 02. “cave” [All Fields] AND “isolation” [All Fields] AND “human” [All Fields], 03. “free running isolation” [All Fields], 04. “potholing” [All Fields], 05. “potholer/s” [All Fields], 06. “caving” [All Fields], 07. “social isolation” [All Fields], 08. “subterranean” [All Fields] AND “isolation” [All Fields], 09. “underground environment” [All Fields], 10. “grotta/e” [All Fields], 11. “isolamento in grotta” [All Fields], 12. “isolamento spazio temporale” [All Fields], 13. “Montalbini” [author], 14. “grotte” [All Fields], 15. “sejours souterrain” [All Fields], 16. “Siffre” [author], 17. “cueva” [All Fields], 18. “aisolamento in cueva” [All Fields], 19. “permanencer bajo tierra” [All Fields], 20. “spelunka” [All Fields], and 21. “spelunking”. Additional manual reviews were performed in specialized speleological databases (see Supplementary Table 1), and forty experts (i.e., seven authors of scientific publications, twenty-one health care providers related to cave/ sports medicine, ten speleologists/ health care providers with direct experience in underground isolation; two journalists and librarians; see Supplementary Table 2) were surveyed/interviewed for additional information.

### Study Records (Selection and Data Collection Process)

Animal studies in the cave environment were excluded from this review. Subjects of any age, gender, medical condition or caving experience were included. Full-length and abstract (i.e., when full-length was not available and the abstract contained original data) peer reviewed articles, as well as other relevant scholarly articles with original data, were considered eligible for inclusion. All retrieved records were screened by title and abstract by one review author (LZ). The review author (LZ) rated each citation using a ”relevant,“ ”irrelevant,“ or ”unsure“ designation. Only retrieved records that received a ”relevant“ or ”unsure“ classification were fully read. These selected articles were then classified into nine different categories (i.e., atmospheric science, cardiovascular function, emergency medicine, human factors, muscle/skeletal system, neuroscience, psychological aspects, radiation and respiratory function), in accordance with relevant topics for space medicine (Videbaek et al., 1993). Only records classified as ”physiological studies“ were included in the current review and two review authors (LZ, GS) independently assessed full-text records (i.e. each record was checked twice). The eligibility assessment was also performed independently, in a standardized manner by two different authors (LZ, GS). Disagreements between reviewers were resolved via discussion and all reasons for study exclusion were recorded.

### Risk of Bias (Quality) Assessment and Data Synthesis

The studies evaluated in this review were further categorized based on the duration of time spent in the cave environment. We classified studies based on cave exposure time: cave exposure up to 72 h as short and above 72 h as prolonged.

Data from included studies were summarized as follows (see Tables 1, 2): the publication details (e.g., publication year, format type, publication status); characteristics of participants (e.g., number, gender, age), and study specifics (e.g., experimental protocol, location of a study and measured parameters). The primary outcomes were defined as the effects of cave exposure on homeostatic control and physiological functions. For the short exposure studies, data about bone composition, cardiovascular, endocrine-metabolic, immunologic, muscular, and visual dysfunction were extracted. In addition, for prolonged exposure studies we extracted measurements related to the rest-activity cycle, thermoregulation, psychomotor function, cardiovascular adaptation, immunologic and hematological changes, bone composition, menstrual cycle, and renal system.

**Table 1:** Characteristics of short exposure studies.

**Author(year)**	**Pinna et al. (2017)**	**Antoni et al. (2017)**	**Lança et al. (2016)**	**Giovine et al. (2009)**	**Maura et al. (2008)**	**Princi et al. (2008)**	**Stenner et al. (2007a)**	**Stenner et al. (2007b)**	**Stenner et al. (2006)**	**Bregani et al. (2005)**	**Stenner (2002)**	**Bratima et al. (1999)**	**Vacca et al. (1994)**
Study design	Longitudinal	Longitudinal	Cross-sectional	Case report	Cross-sectional	Case report	Longitudinal	Longitudinal	Longitudinal	Randomized, double blind, cross-over	Longitudinal	Longitudinal	Longitudinal
Sample size(n) [males]	17 [10]	40 [24]	23 [15]	na	19 [14]	1 [0]	5 [5]	5 [5]	5 [5]	10 [10]	7 [6]	6 [0]	13 [12]
Place (depth/distance)	Cave and lab (−100 m/10.6 ± 2.2^a^ km)	Cave (−100 m/~3 km)	Cave and lab (na/na)	Cave (na/±10 m)	Lab and simulated cave tent	Cave (−500 m/na)	Cave (−700 m/na)	Cave (−700 m/na)	Cave (−700 m/na)	Cave (−350 m/~900 m)	Cave (−770 m/na)	Cave (−700 m/~2–2.5 km)	Caves (Up to −370 m/na)
Environmental temperature (°C)	Cave: 12–15^b^ Lab: 22	14–15^b^	na	47	22	na	2	2	2	na	na	na	na
Humidity (%)	Lab: 50 Cave: 95–100^b^	95–100^b^	na	100	76 lab 100 tent	na	High humidity	High humidity	na	na	na	na	na
Light exposure (head lamps)	na	Yes	Yes	Yes	na	na	na	na	Yes	Yes	na	Yes	na
Time period in cave environment(h)	9.4 ± 1.2^a^	8–10^b^	~6	0.15–1^b^	<1	~23	~20	~20	18	12	~22	up to 6	30 days with cave activity
Exercise	Yes	Yes	Yes	Yes	Yes	Yes	Yes	Yes	Yes	Yes	Yes	Yes	Yes
Interval measurement	Continuous monitoring during cave progression	BL (time 0), continuous monitoring and time end	One evaluation in lab and one evaluation in cave	BL (time 0), time end	BL (time 0), continuous monitoring and time end	BL (time 0), continuous monitoring	Four days before, BL (time 0), time 12, 24, 48 h	BL (time 0), time 12, 24, 48 h	BL (time 0), 1.7, 5, 18 and 24 h	BL (time 0), various time points in cave, and time end	BL (time 0), various time points in cave	BL (time 0), continuous monitoring	BL (time 0) and time end
Parameters	AT, EE,HR, $\overset{∙}{V}$O_2_ peak, Wmax,	BMI, EE, hydration evaluation, nutritional habits, %FFM, %FM,	BVA, contrast sensitivity, intraocular pressure, near visual activity, ocular alignment refractive error, VA	BF, BP, ECG glycemia, HR, SaO_2_, Tcore, urine analysis, visual analog rating scale, Wassler's test	BP, glycemia, HR, SaO_2_, symptoms, la_b_	ECG, HRV, tachogram	Cortisol, FT3, FT4, GH, TSH, %ΔPV	Hb, MCV, RBC, Trectal, %ΔPV	CK, CK-MB, Hp, LDH, leucocytes platelets	BP, CK, creatinine, ECG, electrolytes (Na+,K+, Mg++), HCT, HR, plasma LDH, pH and ketones in urine, urine density	Glycemia, food diary	BMI, FEV1, FVC,HR, Livi index, MVV, skin folds, Tiffeneau-Pinelli index,V'O_2_, %FFM, %FM	Skin folds, TBW, %FFM, %FM,
Other parameters	na	ECG, height, weight, BP, $\overset{∙}{V}$O_2_peak, Wmax,	na	BMI, symptoms, weight	ECG	na	Height, weight, %FM	Height, weight, %FM	Height, weight, skin folds, BMI, %FM	Height, weight, BMI	Height, weight, BMI	Height	Height, weight

a *Mean ± Standard deviation*.

b *Range*.

*AT, anaerobic threshold; BF, breath frequency; BL, baseline; BMI, Body Mass Index; BP, blood pressure; BVA, binocular visual acuity; CK, creatine kinase; CK-MB, MB isoenzyme of CK; ECG, electrocardiogram; EE, energy expenditure; FEV1, forced expiratory volume in the first second; FT3, free triiodothyronine; FT4, free thyroxine; FVC, forced expiratory volume; GH, serum growth hormone; Hb, hemoglobin; HCT, hematocrit; Hp, haptoglobin; HR, heart rate; HRV, heart rate variability; K+, plasma potassium; la_b_, blood lactate; LDH, lactate dehydrogenase; MCV, mean corpuscular volume; Mg++, plasma magnesium; MVV, maximal voluntary ventilation; na, not available; Na+, plasma sodium; RBC, red blood cell; SaO_2_, oxygen saturation of hemoglobin; TBW, total body water; Tcore, body core temperature; Trectal, rectal temperature; TSH, thyroid-stimulating hormone; VA, distance visual acuity; $\overset{∙}{V}O_{\text{2}}$ peak, peak oxygen uptake; Wmax, maximum workload; %FFM, fat free mass percentage; %FM, fat mass percentage; %ΔPV, percentage changes of plasma volume*.

**Table 2:** Characteristics of prolonged exposure studies.

**Author(year)**	**Cornelissen et al. (2010)**	**Hillman et al. (1994b)**	**Hillman et al. (1994a)**	**Sonnenfeld et al. (1992)**	**De la Pena et al. (1989)**	**Chouvet et al. (1974)**	**Mills et al. (1974)**	**Halberg et al. (1970)**	**Oleron et al. (1970)**	**Apfelbaum et al. (1969)**	**Ghata et al. (1969)**	**Colin et al. (1968)**	**Fraisse et al. (1968)**	**Siffre et al. (1966)**	**Reinberg et al. (1966)**	**Halberg et al. (1965)**	**Mills (1964)**	**Kleitman (1963)**
Study design	Longitudinal	Longitudinal	Longitudinal	Longitudinal	Longitudinal	Longitudinal	Longitudinal	Longitudinal	Longitudinal	Longitudinal	Longitudinal	Longitudinal	Longitudinal	Longitudinal	Longitudinal	Longitudinal	Longitudinal	Longitudinal
Sample size (*n*) [males]	1 [0]	1 [0]	1 [0]	1 [0]	1 [0]	3 [3]	1 [1]	2 [1]	1 [1]	7 [0]	2 [1]	1 [1]	2 [2]	2 [1]	1 [0]	1 [1]	1 [1]	2 [2]
Subject's age(years)	28	32	32	27	27	23; 23; 28	28	M: 35F: 25	24	20–35^b^	M: 35 F: 25	25	na	M: 35F: 25	26	na	na	20;43
Place (depth)	Lab in cave!!!!!—special habitat like Lunar Base (na)	Cave (−80 m)	Cave (−80 m)	Lab in cave (−9.1 5 m)	Cave (na)	Different caves (−70 m)	Cave (na)	Two different caves with tent, tables, chairs, instruments(−80; −7 0 m)	Cave (−70 m)	Cave with tents, tables, chairs, instruments (−100 m)	Two different caves with tents, beds, chairs (−80; −70 m)	Cave with tent (−70 m)	Cave (na)	Two different caves with tents, beds, chairs (−80; −7 0 m)	Cave (na)	Cave (−130 m)	Cave (na)	Cave with habitat, tables, beds, chairs (~−45 m)
Environmental temperature(°C)	na	na	na	na	22 ± 0^a^	6	na	6 ± 2^a^	na	11 ± 0.1 ^a^	6 ± 2 ^a^	Cave: 6–7^b^Tent: 20	na	6 ± 2 ^a^	na	na	7	12
Humidity(%)	na	na	na	na	90	100	na	100	na	98	100	100	na	100	na	na	~100	na
Light exposure (artificial light)	yes	yes	Yes	Yes, constant illuminated	na	Yes, constant illuminated	na	Yes, lamps 50 lux	na	Yes, light by helmet5–10^b^ lux	Yes, as desired	Yes	na	Yes, lamps 50 lux	Yes, light 50 lux	Yes, pleasure ~ 13.5 lm	Yes, candles or forehead lamp	Yes, lanterns
Isolation (way of communication**)**	Yes (na)	Yes (telephone)	Yes (telephone)	Yes (computer)	Yes (computer)	Yes (telephone, no clock)	Yes (telephone, no clock)	Yes (telephone, no clock)	Yes (telephone, no time estimation)	Yes (telephone, no time estimation)	Yes (telephone, no time estimation)	Yes (telephone, no time estimation)	Yes (telephone, no time estimation)	Yes (telephone, no time estimation)	Yes (no time estimation)	Yes (telephone, no clock)	Yes!!!!!i (telephone and yes clock)	Yes (na)
Time periodin cave environment(days)	267	103	103	131	97	174;150;150	127	Male: 125 Female: 88	174	14	Male: 125 Female: 88	174	58; 174	Male: 125 Female: 88	88	62	105	32
Exercise	na	na	na	na	na	Yes	na	yes	na	na	na	yes	na	na	na	na	Yes	na
Interval measurement	24 days before, continuous (20 min), 69 days after	Before, during and after isolation	Before, during and after isolation	Before, during and after isolation	Various time points during isolation	5 days before, during and 5 days after isolation	During and after isolation	Before, during and after 1 month of isolation	During and after isolation	Before, during and after isolation	Before, during and 1 month after isolation	Various time points during isolation	Various time points during isolation	Before, during and after isolation	Before, during and after isolation	Various time points during isolation	One day before, during and after isolation	Various time points during isolation
Parameters	BP cycle, HR cycle	Caffeine metabolite ratio, creatinine excretion rate, Turinary, urinary volume, 17-OHCS	Caffeine metabolite ratio, estimation period time sleep-wake cycle, urinary system cycle, Turinary, water excretion rate, 17-OHCS	Hb, HCT, IFN-γ, leucocytes, lymphocytes, MCH, MCHC, MCV, NK, platelet, RBC, WBC	BP cycle, HR cycle, Taxillary Tsublingual	EEG, EMG, EOG	Chloride cycle, creatine, KU, NaU, phosphate, sleep-wake cycle, plasma steroid	Estimation period time, HR cycle, KU, Trectal, urinaryvolume, 17-OHCS	Estimation of spontaneous time and reaction time, sleep-wake cycle	Sleep-wake cycle	Sleep-wake cycle, Trectal, 17-OHCS	Sleep-wake cycle, spontaneous activity,Trectal	Estimation period time, simple reaction time, sleep-wake cycle	Blood values, HR cycle, oftalgic parameters, sleep-wake cycle, Trectal, urinary volume	Menstrual cycle, Trectal	Estimation period time, HR cycle, sleep-wake cycle	Creatinine, electrolytes (chloride, NaU, KU), urinary pH cycle, sleep-wake cycle	Sleep-wake cycle, Tcore
Other parameters	Cosmic ray	Cosmic ray, menstrual cycle	Defecation, drinking eating	Bone density, BP cycle, menstrual cycle, sleep-wake cycle	na	Cardiac cycle, sleep-wake cycle, Trectal	na	Grip strength, menstrual cycle, sleep-wake cycle, visual evaluation, weight	Meals cycle	na	na	EEG, sleep-wake cycle	na	Dynamometric force tests, K+, Na+, weight	na	na	Steroid estimation	na

a *Mean ± Standard Deviation*.

b *Range*.

*BP, blood pressure; EEG, electroencephalogram; EMG, electromyography; EOG, electrooculography; HCT, hematocrit; Hb, hemoglobin; HR, heart rate; IFN-γ, interferon gamma; K+, plasma potassium; MCH, mean corpuscular hemoglobin; MCHC, mean corpuscular hemoglobin concentration; MCV, mean corpuscular volume; na, not available; Na+, plasma sodium; NK, natural killer cells; RBC, red blood cells; Tcore, core body temperature; Turinary, urinary temperature; Trectal, rectal temperature; KU, urinary potassium; NaU, urinary sodium; WBC, white blood cells; 17-OHCS, 17-hydroxycorticosteroid*.

## Results

### Study Selection

**Figure 1:**
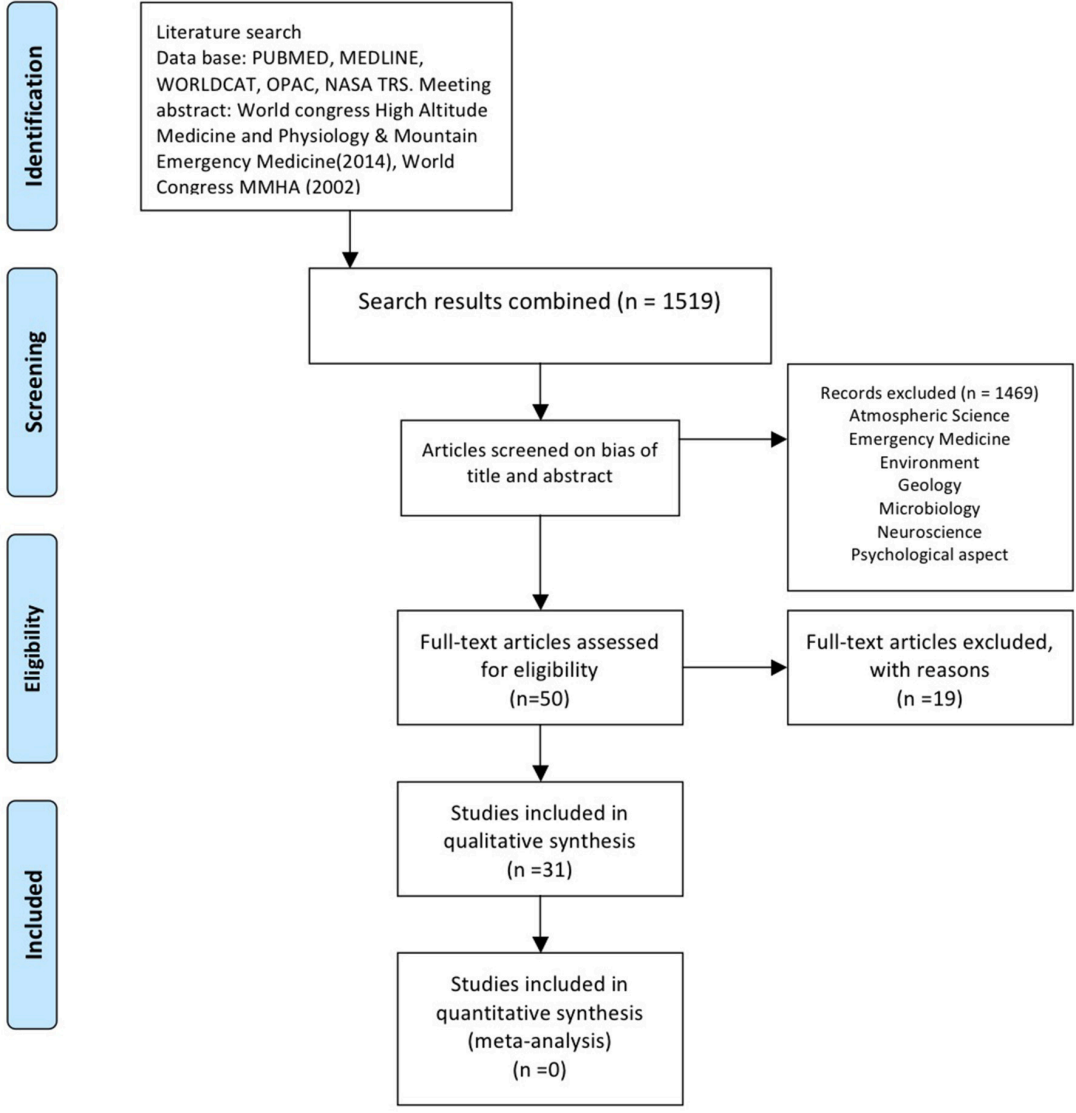
Search and screening strategy based on PRISMA guidelines.

The systematic search (including manual review and expert suggestions) retrieved 1,519 articles (Figure 1). After the initial screening, 50 articles were retained; 31 of these were classified as ”physiological studies.“ A list of (and reasons for) the 19 excluded articles is recorded in Figure 1 and Supplementary Table 3.

### Study Characteristics

Thirteen papers were categorized on the short exposure effects and 18 on prolonged exposure. The characteristics of those studies considered eligible for inclusion are summarized in Tables 1, 2, respectively.

All 31 studies included in the review contained original data and were published in either English, Italian or French. The studies differed in study design (e.g., time exposure, exercise, isolation and diet), cave environment characteristics (e.g., temperature, altitude and artificial light/environment exposure), monitored parameters, and measurement interval. The marked methodological heterogeneity across these studies and the limited number of papers prevented meta-analysis. Hence, we continue to describe these results qualitatively.

### Short Exposure Effect Studies

These 13 studies, all published between 1994 and 2017, include a total of 151 participants in different cave/lab simulation settings. Cave/lab simulation exercises were performed in all studies; exercise can be classified as cave progression (characterized by atypical, strenuous movement, often combining ascent, descent, and scrambling activity) in all studies apart from one, where an exercise stress test in the simulated cave-environment condition was completed (Maura et al., 2008). Anthropometric characteristics were measured in the majority of the studies (Vacca et al., 1994; Bratima et al., 1999; Stenner, 2002; Bregani et al., 2005; Stenner et al., 2006, 2007a,b; Maura et al., 2008; Antoni et al., 2017; Pinna et al., 2017). Overall, study subjects were healthy with heterogeneous anthropometric characteristics. Cardiovascular, endocrine-metabolic, immunologic-hematological, muscular responses, and visual dysfunction were investigated (Table 1). The corresponding trend analysis of the results for all studies can be found detailed in Table 3.

**Table 3:** Short exposure studies main results.

**Author (year)**	**Pinna et al. (2017)**	**Antoni et al. (2017)**	**Lança et al. (2016)**	**Giovine et al. (2009)**	**Maura et al. (2008)**	**Princi et al. (2008)**	**Stenner et al. (2007a)**	**Stenner et al. (2007b)**	**Stenner et al. (2006)**	**Bregani et al. (2005)**	**Stenner (2002)**	**Bratima et al. (1999)**	**Vacca et al. (1994)**
V°O_2_ (ml^.^kg^−1.^min^−1^)	Peak in lab 32.4 ± 6.4^a^	Peak in lab 34.6 ± 3.4^a^	na	na	na	na	na	na	na	na	na	22–46^b^	na
EE (MET's)	na	4.1 ± 0.7^a^ (M) 4.1 ± 0.5^a^ (F)	na	na	na	na	na	na	na	na	na	na	na
EE (kcal^.^h^−1^)	In lab at exhaustion: 674.3 ± 197.7^a^ In cave: 268.8 ± 54.8^a^	287.5 ± 48.5^a^ (M) 225.4 ± 27.9^a^ (F)	na	na	na	na	na	na	na	na	na	na	na
FFM (%)	na	na	na	na	na	na	na	na	na	na	na	71.80–81.56^b^	↓
FM (%)	na	=	na	na	na	na	20.4 ± 0.8^a^	19.1–21.4^b^	19.1–21.4^b^	na	na	18.44–28.20^b^	↓
SaO_2_!!!!! (%)	na	na	na	na	90–95^b^	na	na	na	na	na	na	na	na
Tcore (°C)	na	na	na	↑	na	na	na	↑	na	na	na	na	na
BP (mmHg)	na	na	na	↑	=	na	na	na	na	↑	na	na	na
HR (beats^.^min^−1^)	In lab: 166 ± 8.5^a^	na	na	↑	↑	na	na	na	na	↑	na	↑	na
LF/HF	na	na	na	na	na	↑	na	na	Na	na	na	na	na
CK (U^.^l^−1^)	na	na	na	na	na	na	na	na	↑	↑	na	na	na
LDH (U^.^l^−1^)	na	na	na	na	na	na	na	na	↑	↑	na	na	na
Hp (g^.^l^−1^)	na	na	na	na	na	na	na	na	↓	na	na	na	na
La_b_ (mmol^.^l^−1^)	na	na	na	na	Normal air: 4.22 ± 1.39^a^ Rarefied air: 3.58 ± 1.45 ^a^	na	na	na	na	na	na	na	na
GH (μg^.^l^−1^)	na	na	na	na	na	na	↑	na	na	na	na	na	na
Cortisol (mmol^.^l^−1^)	na	na	na	na	na	na	↓	na	na	na	na	na	na
FT3 (pmol^.^l^−1^)	na	na	na	na	na	na	=	na	na	na	na	na	na
FT4 (pmol^.^l^−1^)	na	na	na	na	na	na	↑	na	na	na	na	na	na
TSH (μlU^.^ml^−1^)	na	na	na	na	na	na	=	na	na	na	na	na	na
Glycemia (mg^.^dl^−1^)	na	na	na	na	Normal air: 85.5 ± 13.57 ^a^ Rarefied air: 90.57 ± 14.19 ^a^	na	na	na	na	na	~80	na	na
RBC (10^12.^l^−1^)	na	na	na	na	na	na	na	↑	na	na	na	na	na
Hb (g^.^l^−1^)	na	na	na	na	na	na	na	↑	na	na	na	na	na
MCV (fL)	na	na	na	na	na	na	na	↓	na	na	na	na	na
ΔPV (%)	na	na	na	na	na	na	↓	↓	na	na	na	na	na
Lymphocytes (10^3^ μL)	na	na	na	na	na	na	na	na	↓	na	na	na	na
Leucocytes (10^3^ μL)	na	na	na	na	na	na	na	na	↑	na	na	na	na
Neutrophils (10^3^ μL)	na	na	na	na	na	na	na	na	↑	na	na	na	na
Monocytes (10^3^ μL)	na	na	na	na	na	na	na	na	↑	na	na	na	na
Platelets (10^3^ μL)	na	na	na	na	na	na	na	na	↑	na	na	na	na
Visual performance	na	na	BVA = contrast sensitivity ↑	na	na	na	na	na	na	na	na	na	na
Symptoms	na	na	No visual pathologies related to cave environment	Dyspnea, heat stroke, weakness (device failure)	Headache, heat sensation dizziness, ocular pruritus, trembling hands	na	na	na	na	Better recovery from efforts	na	na	na

a Mean ± Standard Deviation

b Range; ↑, increased; ↓, decreased; =, stable

*BP, blood pressure; BVA, binocular visual acuity; CK, creatine kinase; EE, energy expenditure; FFM, fat free mass; FM, fat mass; FT3, free triiodothyronine; FT4, free thyroxine; GH, growth hormone; Hb, hemoglobin; Hp, haptoglobin; HR, heart rate; la_b_, blood lactate concentration; LDH, lactate dehydrogenase; LF/HF, low frequency and high frequency power spectral density ratio; MCV, mean corpuscular volume; RBC, red blood cells; SaO_2_, oxygen saturation of hemoglobin; Tcore, body core temperature; TSH, thyroid-stimulating hormone; $\overset{∙}{V}O_{2}$ oxygen uptake; ΔPV, changes of plasma volume*.

#### Cardiovascular Changes

One study (Bratima et al., 1999) reported that heart rate (HR) increased more during cave traverse (ascent and scrambling activity) compared to descent into the cave. Other studies have not been able to substantiate these findings either pre or post, reporting only a trend for increased HR during cave exploration, without presenting sufficient data to that effect (Vacca et al., 1994; Giovine et al., 2009). Notably, a later study by Maura et al. (2008) used a unique scientific approach, directly studying the environmental factors that may be definitive in HR modification, in a specially built subterranean laboratory. This study demonstrated that in comparison to normoxic conditions, HR was on average increased by 10 beats^.^min^−1^ (*p* < 0.0002), when cavers performed a constant work rate exercise on a cycle ergometer at 75% of their maximum theoretical heart rate in an hypoxic (O_2_ ~15%) hypercapnic (CO_2_ ~1.9%) humid (100%) simulated cave atmosphere (Maura et al., 2008). These results were corroborated further in a later case report, detailing a cave exploration under similar conditions, which showed a relevant modification of the cardiac autonomic nervous system (ANS) activity through a reduction in heart rate variability (HRV) and a shift in the sympathovagal balance toward more sympathetic and less parasympathetic activity in a female elite potholer (Princi et al., 2008).

#### Endocrine and Metabolic Changes

Physiological/psychological responses during cave progression have been characterized by indicative stressors considered able to alter the hormonal balance and metabolic homeostasis in humans. Previous work focused on hypothalamus-pituitary adrenocortical, hypothalamus-pituitary and hypothalamus-pituitary thyroid system responses to duration cave activity of _~_20 h. Results demonstrated an evident elevation of serum cortisol prior to cave entry, even in elite cavers. This initial anticipatory stress reaction was reportedly reduced over time, followed by a noticeable increase in growth hormone (GH) production during prolonged explorative cave activity (Stenner et al., 2007a). Recently, Antoni et al. (2017) reported that total energy expenditure (TEE) was in the range 225–287 kcal.h^−1^ for women and men for a standardized cave progression (i.e., 10 h subterranean exploration), which is equivalent to an average metabolic equivalent of task (METs) of 4.1 METs. Energy intake during this cave exploration (determined by means of a self-administered dietary recall) was calculated to be ~100–120 kcal. h^−1^ (Antoni et al., 2017), this value was also supported by previous work in this field (Stenner, 2002). Bayesian statistical analysis estimated the effect of predictive variables on TEE, revealing that experienced cavers had a 5% lower TEE than those less skilled, plus female participants required a comparatively larger EE than men to perform the same task. Previous work has shown a significant decrease in fat mass estimated by skin folds and bio-impedance after repeated daily cave progression (Vacca et al., 1994), however as expected no change in fat mass was reported after only 10 h of cave exploration (Antoni et al., 2017). Glycemic values were shown to be in the normal range both during a 22 h continuous cave progression (Stenner, 2002) and during stress testing (≤ 1h) in a simulated cave environment (Maura et al., 2008).

#### Immunological and Hematological Changes

A previous study demonstrated an initial increase in red blood cell (RBC) count and hemoglobin (Hb) concentration after only ~5 h of cave exploration, although this increase was in parallel with a concomitant reduction in plasma volume (PV) (Stenner et al., 2007b). Previous work from the same research group demonstrated that the number of leucocytes, neutrophils, monocytes, and platelets were significantly increased after cave progression, whereas lymphocytes showed a concomitant significant reduction (Stenner et al., 2006).

#### Muscular Changes

Two studies showed an increase in serum markers for muscle damage and metabolism during cave progression (Bregani et al., 2005; Stenner et al., 2006). Specifically, there was a clear increase in serum creatine kinase (CK) and lactate dehydrogenase (LDH) (Bregani et al., 2005; Stenner et al., 2006). Haptoglobin values were also significantly decreased (Stenner et al., 2006). The effect of specific nutritional supplementation of creatine and branched-chain amino acids administration after cave progression was investigated in a cross-over, randomized, double blind study (Bregani et al., 2005). Participants treated with creatine showed lower levels of CK and LDH after cave progression in relation to the control group. The LDH increase after exercise was more pronounced in controls, even if it was not significant; post cave exercise, CK values were increased significantly, but less so in supplemented participants.

#### Visual System Changes

No signs or symptoms of visual dysfunctions where reported post intermittent exposure to the cave environment in a recent study (Lança et al., 2016). Binocular visual acuity (BVA) was reported to be −0.05 ± 0.15 LogMAR (20/18) with a head light with a mean illuminance of 451.0 ± 305.7 lux. Significant improvements in contrast sensitivity were observed with 450 nm filters for 6 cycles per degree (cpd) and 18 cpd spatial frequencies.

### Prolonged Exposure Effect Studies

The 18 studies included within this review that were related to prolonged exposure to the cave environment were published between 1963 and 2010. In total, all except one of these prolonged exposure studies exposed participants directly to the cave environment for >30 days (Table 2). Specifically, in Sonnenfeld et al. (1992) and Cornellissen at al. (2010), participants were accommodated within a laboratory built inside a cave, while in the others there was no such facility. Overall, in all 18 studies, participants were healthy and in a number of articles under review, the same participants were recruited repeatedly. In total, 20 participants were studied, with a good proportion of male and female (nine and 11, respectively).

Those studies inclusive of an isolation component mainly investigated whether biological rhythms persist or desist in the absence of standard environmental conditions (e.g., without information related to the 24 h clock). During underground isolation, participants were asked to follow a regular wake-sleep cycle, including customary meal times in the absence of any scheduling cues (Mills, 1964; Mills et al., 1974) (Table 2). Corresponding trend analysis of results for all studies can be found detailed in Table 4.

**Table 4:** Prolonged exposure studies main results.

**Author** **(year)**	**Cornelissen et al. (2010)**	**Hillman et al. (1994b)**	**Hillman et al. (1994a)**	**Sonnenfeld et al. (1992)**	**De la Pena et al. (1989)**	**Chouvet et al. (1974)**	**Mills et al. (1974)**	**Halberg et al. (1970)**	**Oleron et al. (1970)**	**Apfelbaum et al. (1969)**	**Ghata et al. (1969)**	**Colin et al. (1968)**	**Fraisse et al. (1968)**	**Siffre et al. (1966)**	**Reinberg et al. (1966)**	**Halberg et al. (1965)**	**Mills (1964)**	**Kleitman (1963)**
Sleep-wake cycle	na	na	↑	↑	na	↑	↑	↑	↑	↑	↑	↑	↑	↑	na	↑	↑	Imposed 28 h One sub. ↑ Older sub. =
Amplitude (cycle period)	na	na	↑	↑	na	↑	↑	na	↑	↑	↑	↑	↑	↑	na	↑	↑	na
Thermoregulatory cycle	na	↑	↑	na	↑	↑ (only for one subject)	na	↑	na	na	↑	= (circadian 24 h)	na	= (circadian 24 h)	↑	na	na	= (~circadian 24 h)
Amplitude (cycle period)	na	↑	↑	na	↑	na	na	↑	na	na	↑	↑	na	↑	↑	na	na	na
Cardiorespiratory cycle	↑	na	na	na	↑	na	na	na	na	na	na	na	na	na	na	na	na	na
BP cycle	↑	na	na	7 day circaseptan rhythm	↑	na	na	na	na	na	na	na	na	na	na	na	na	na
HR cycle	↑	na	na	na	↑	na	na	na	na	na	na	na	na	Circadian 24 h(↑ period)	na	↑	na	na
Renal cycle	na	↑	↑	na	na	na	↑	na	na	na	na	na	na	na	na	na	↑	na
Water excretion rate	na	↑	↑	na	na	na	na	na	na	na	na	na	na	na	na	na	na	na
NaU cycle	na	na	na	na	na	na	=	na	na	na	na	na	na	na	na	na	Irregular	na
KU cycle	na	na	na	na	na	na	=	Irregular	na	na	na	na	na	na	na	na	↑	na
17-OHCS cycle	na	↑	↑	na	na	na	na	↑	na	na	↑	na	na	na	na	na	na	na
Menstrual cycle	na	↑	na	Halted in the first month	na	na	na	na	na	na	na	na	na	na	↓	na	na	na
RBC(10^12.^l^−1^)	na	na	na	↓ =	na	na	na	na	na	na	na	na	na	=	na	na	na	na
Hb(g^.^l^−1^)	na	na	na	↓ =	na	na	na	na	na	na	na	na	na	=	na	na	na	na
HCT	na	na	na	↓↑	na	na	na	na	na	na	na	na	na	na	na	na	na	na
Leucocytes!!!!!(10^3^μL)	na	na	na	↓	na	na	na	na	na	na	na	na	na	=	na	na	na	na
Lymphocytes (10^3^ μL)	na	na	na	↑	na	na	na	na	na	na	na	na	na	na	na	na	na	na
Platelets (10^3^)	na	na	na	↓↑	na	na	na	na	na	na	na	na	na	na	na	na	na	na
Visual performance	na	na	na	na	na	na	na	Transient modification Speed of chromatic vision/ Binocular vision	na	na	na	na	na	= apart from chromatic perception	na	na	na	na
Bone density (%)	na	na	na	↓	na	na	na	na	na	na	na	na	na	na	na	na	na	na
Simple reaction time	na	na	na	na	na	na	na	na	↑↓	na	na	na	↑	na	na	na	na	na
Estimation period time	na	na	↑	na	na	na	na	↓	↑	na	na	na	= ↓↑	na	na	↑	na	na
Symptoms	na	na	na	na	na	na	na	na	na	na	na	na	na	Diarrhea	na	na	na	na

a *Mean ± Standard Deviation*.

b *Range; ↑, increased; ↓, decreased; = , stable*.

*BP, blood pressure; Hb, hemoglobin; HCT, hematocrit; HR, heart rate; KU, urinary potassium; NaU, urinary sodium; RBC, red blood cells; sub., subjects; 17-OHCS, 17-hydroxycorticosteroid*.

#### Rest-Activity Cycle

The alterations observed in individual rest-activity cycles affected all physiological variables considered in the studies under review. A rest-activity cycle persisted in the absence of any environmental synchronizer or deliberate scheduling, however it appeared desynchronized from an exact 24 h period in all the 14 studies included in review where it was investigated (Kleitman, 1963; Mills, 1964; Halberg et al., 1965, 1970; Siffre et al., 1966; Colin et al., 1968; Fraisse et al., 1968; Apfelbaum et al., 1969; Ghata et al., 1969; Oleron et al., 1970; Chouvet et al., 1974; Mills et al., 1974; Sonnenfeld et al., 1992; Hillman et al., 1994a).

Colin et al. (1968) observed that at the beginning of their 6-month isolation case study, both the actual and estimated rest-activity cycles of their single male participant was near circadian (25 h and 27 h, respectively). After only 10 days of isolation, the participant reached values of 45 h referring to the time between two awakenings and 50 h referring to the estimated period of activity. The estimated period of activity progressively increased (bicircadian), while the wakening-to-wakening cycles returned to circadian (Colin et al., 1968). Later, a further study with a similarly long duration isolation (Chouvet et al., 1974) also showed similar discrepancies between the actual and the estimated wakening-to-wakening cycles, reporting data from polygraphic recordings [i.e., electroencephalographic activities (EEG) and electromyography (EMG)] in three participants with variable latencies and several variations in sleep observations. All participants reached a bicircadian rhythm (34 h of wakefulness followed by 14 h of sleep), which they subjectively considered to be a 24 h cycle. However, only one participant was able to adapt completely to this new rhythm and maintain it >2 months. The use of an external Zeitgeber (i.e., 500 W lamp) was effective in reducing the relative variability of this rhythm. Paradoxical sleep (PS; also known as rapid eye movement or REM sleep) remained unchanged while the sleep-waking rhythm was disrupted in all, but PS onset latencies in the two participants unable to maintain a bicircadian rhythm were shortened (Chouvet et al., 1974). Hillman et al. (1994a), showed that a female participant at the start of 103 days of isolation demonstrated an immediate change in rest-activity cycle to ~25 h period. In the middle of the span, this period reportedly shortened slightly but still remained longer than 24 h. In addition, the participant showed a dissociation between observed and estimated daily duration, averaging ~58 h per subjective ”day.“ Differences between the perceived time elapsed and the actual time elapsed (calendar days) was found also by Halberg et al. (1970), indicating that the two participants both perceived the day to be longer than normal, over the course of the isolation period (88–128 days).

Similar trends were also previously observed in long duration isolation studies (Fraisse et al., 1968; Mills et al., 1974), where different male participants also reported dissociation between observed and estimated cycle duration. More specifically, Mills et al. (1974) demonstrated in a male participant isolated for a total of 4 months (127 days) an extended estimated day length adopted by the subject, with an estimated activity period ranging from ~19 to 66 h (Mills et al., 1974). Previously, Mills demonstrated that the sleep-activity cycle was considered externally desynchronized. However, the internal synchronization reportedly persisted into a rhythmic process, with a period of ~24.5 h (Mills, 1964). Finally, Halberg et al. (1965) showed that a 2-month isolation in a cave environment, despite the desynchronization with respect to local time, an internal synchronization of the rhythm functions was maintained (Halberg et al., 1965). Overall an internal rhythm was maintained, without the influence of environmental synchronizers, with different period not based on the 24 h (and for this reason is called free-running).

#### Thermoregulatory System

The circadian rhythm of the body temperature was investigated in 10 studies (Kleitman, 1963; Reinberg et al., 1966; Siffre et al., 1966; Colin et al., 1968; Ghata et al., 1969; Halberg et al., 1970; Chouvet et al., 1974; De la Pena et al., 1989; Hillman et al., 1994a,b). The first underground study investigating the rhythm of body temperature, Kleitman (1963), revealed a persistence of a circadian rhythm for a month in a participant living on a forced-desynchrony protocol (i.e., non 24 h sleep/wake cycle, with no regular light stimulus). In contrast to the work by Kleitman (1963), Colin et al. (1968) compared the rhythms of body temperature and spontaneous activity for a 6-month isolation period, observing maintenance of a circadian rhythm of the rectal temperature throughout the duration of isolation with a progressive lengthening. However, in an earlier study, over the course of ~3 months subterranean isolation rectal temperature decreased with circadian desynchronization compared to local time following a circatrigintan rhythm (i.e., a biologic variation or rhythm with a frequency of 1 cycle in 30 ± 5 days) (Reinberg et al., 1966). There was a similar observation in later study, in which an internal circasemiseptan rhythm desynchronization (relating to biologic variations or rhythms with a frequency of 1 cycle in 3.5 ± 1 days) was observed in urinary-temperature (Hillman et al., 1994b).

#### Psychomotor Tests

Several studies reported data related to psychomotor tests performed by subjects during underground isolation (Halberg et al., 1965, 1970; Fraisse et al., 1968; Oleron et al., 1970; Hillman et al., 1994a). Estimation of a short interval of time (i.e., number of seconds the subject took to count from 1 to 120 s with the goal of evaluating a duration of 2 min) showed a progressive increase within a 2-month cave isolation (Halberg et al., 1965). Oleron et al. (1970) and Fraisse et al. (1968), reporting results from one subject, showed an accelerated spontaneous time at the end of the isolation period. During these studies, the participant was required to tap as regularly as possible on a telegraphic key with their middle finger only. The number of taps per 10 s was calculated, plus the interval duration between two taps on average. Moreover, the simple reaction time (i.e., subject was asked to press as quickly as possible on a telegraph key at the appearance of a light signal) was reportedly increased during a prolonged stay in the cave environment.

#### Cardiovascular Changes

HR values and spectrum analysis were covered by four of the studies under review (Halberg et al., 1965; Siffre et al., 1966; De la Pena et al., 1989; Cornelissen et al., 2010). In all of these studies, the HR cycle was desynchronized from environmental synchronizers (i.e., alternation of light-dark cycle, seasons, and social routine), which help to maintain a normal circadian rhythm of ~24 h. HR spectrum analysis during a free-running isolation experience (Siffre et al., 1966) showed that the circadian rhythm persisted with a slight lengthening (24.8 and 24.6 h for the male and female subject, respectively). The three other studies supported this proposition for a persistent circadian drive, regardless of external desynchronization (Halberg et al., 1965; De la Pena et al., 1989; Cornelissen et al., 2010). Equally, similar results were found for blood pressure (BP) spectrum analysis (De la Pena et al., 1989; Cornelissen et al., 2010).

#### Immunologic and Hematological Changes

Only two studies investigated the immunologic and hematological responses due to underground isolation (Siffre et al., 1966; Sonnenfeld et al., 1992). Sonnenfeld et al. (1992) demonstrated in a female a decrease in leukocytes and concomitant increase in both lymphocytes and monocytes, including natural killer T-cells, during a prolonged isolation (131 days). After an initial decrease in RBC, Hb, platelets and hematocrit (HCT) after two weeks, RBC and Hb values reportedly normalized, whereas HCT and platelets increased over and after the isolation period (4 months). However, these results are in direct conflict with the observations in two subjects of Siffre et al. (1966), which demonstrated no changes in RBC, Hb, and leucocytes after an isolation period of either 2 or 4 months.

#### Bone and Mineral Changes

One study reported an overall bone loss of 2% (up to 8.2% in trabecular bone) after 131 days of cave isolation in a woman with a vitamin D-depleted diet (Sonnenfeld et al., 1992).

#### Menstrual Cycle

Changes in the menstrual cycle have been studied in two different women during long isolation in a cave (Reinberg et al., 1966; Hillman et al., 1994b). The menstrual cycle was slightly reduced (from 29 to 26 days) during the isolation period of 88 days (Reinberg et al., 1966). Opposite results, with a lengthening and desynchronization of the menstrual cycle (from 25 to 27–32 days) were also observed in a similar 3-month isolation study (Hillman et al., 1994b).

#### Renal System

Seven studies investigated parameters of renal function during cave isolation (Mills, 1964; Siffre et al., 1966; Ghata et al., 1969; Halberg et al., 1970; Mills et al., 1974; Hillman et al., 1994a,b). During a long permanence in a cave (≤ 4 months), the biological rhythm of urinary excretion of 17-Hydroxycorticosteroids persisted with a desynchronization from circadian rhythm (24 h) (Ghata et al., 1969). Equally, after a similar prolonged isolation study (3 months), the excretion of potassium was also shown to be irregular (Halberg et al., 1970). Mills (1964) in their early work supported this finding, demonstrating a desynchronized rhythm of excretion in both potassium and particularly chloride. Urinary pH was roughly correlated with rate of potassium excretion and creatinine excretion pattern was irregular, without definitive conclusions (Mills, 1964). However, in a later publication they concluded the period of circadian excretion of potassium, sodium and chloride remained similar during cave isolation (127 days), demonstrating a potential variability in methodology, or simply a lack of reproducibility of specific study conditions (Mills et al., 1974). Finally, subsequent studies continued to further demonstrate an internal circasemiseptan desynchronization in water excretion rate, intermicturition interval and caffeine metabolite ratio over a 3-month isolation period (Hillman et al., 1994a,b).

## Discussion

Space medicine is akin to sports medicine in that it is commonly described as dealing with normal physiology in abnormal conditions. Despite this reality, knowledge surrounding the provision of successful physiological preparation in subterranean space analog scenarios remains limited. A current proposition for Martian caves and lava tubes as potential locations for sheltered habitation on Mars, have highlighted the need to better understand both the environmental impact this may infer on pioneering space cohorts and inform the type of training necessary to enable further human space exploration. Previous human studies investigating the effect of the unique cave environment on homeostatic physiology fall into two categories: either short or prolonged exposure. Short-term cave exposure studies have shown an augmentation of heart rate, muscular damage, initial anticipatory stress reaction, and inflammatory responses during cave progression. The cave environment seems to lead to supra-elevated energy expenditure, despite the moderate intensity implied, especially if continued for several hours. Prolonged exposure studies mainly investigated whether biological rhythms persist or desist in the absence of standard environmental conditions. Changes were evident in estimated vs. actual rest-activity cycle periods and external desynchronization, body temperature, performance reaction time, and heart rate cycles. Studies have shown a variability related to the different study environments and design (e.g., time period in cave environment, exercise protocol, control of the influence of environmental characteristics). The establishment of the cave environment as a space analog has further focused these studies on both the acute response to cave progression, as well as adaptive mechanisms involved when mid and long duration exposure to altered atmospheric composition, artificial light, isolation, confinement and challenging combinations of heat and high humidity were imposed. As a result, the environmental effect of cave progression, inclusive of alterations in the circadian rhythm, have been identified as important for informing practical considerations when defining implications for astronaut expeditionary training courses in subterranean space analogs. Nevertheless, through systematic review, we have identified knowledge gaps and areas that would benefit from additional research efforts, such as developing specific criteria for the collection of scientific data specifically related to clinical grade physiological monitoring in challenging subterranean environments. This approach could prove popular throughout organizations where highly trained human operatives are exposed to complex, dangerous working conditions, often in remote locations, void of immediate clinical care and evacuation.

### Studies About Short Exposure Effects in Cave Environments (Focusing on Cave Progression)

Cave exploration is an atypical, strenuous, three-dimensional human movement specific to cave progression, with some similarities with mountaineering (i.e., a combination of ascending, descending and scrambling). The cave studies selected for review herein include a number of cave progressions (see Table 3), which typically alternate between aerobic and anaerobic exercise models in individuals who specialized in caving. Antoni et al. estimated an EE during cave progression for up to 10 h with a prevalence of scrambling in agreement with previous work by Bratima et al., which detailed a prevalence of ascending and descending activity during the traverse (Bratima et al., 1999; Antoni et al., 2017). Nevertheless, similar to a number of case studies including long duration cave progression, there is a lack of detailed analysis of the traverse obstacles or segmentation of the cave into different logistical categories. Current study results pertaining to estimated EE (as well as METs) have shown that a standardized cave traverse can be considered to elicit an energy expenditure equivalent to moderate physical activity (Ainsworth et al., 2000): comparable to modest intensity activities such as fishing, hunting and visiting natural environments recreationally (Ainsworth et al., 2000; Elliott et al., 2015). Despite this, it should be kept in mind that cave explorations are usually carried out for prolonged periods of time, under atypical atmospheric and temperature conditions. This type of prolonged physical effort, despite the moderate intensity implied, could lead to supra-elevated energy expenditure, which should be considered during space exploration analog training models. Previous research regarding Extravehicular Activity (EVA) during Shuttle and ISS missions, as well as in Lunar analogs, seem to be characterized by a very low to moderate metabolic demand and several hours of activity (Johnston et al., 1975; Downs et al., 2018). The estimated EE of 4.1 METs reported during caving activity can be compared to the amount of oxygen required for 28 Apollo EVA (i.e., mean $\overset{∙}{V}$O_2_ = 10–14 ml^.^kg^−1.^min^−1^) (Johnston et al., 1975). Moreover, “Martian” surface traverse (i.e., 1,500 m track covered with rocks and pebbles) and hill climbing, movements typically present during a cave progression, were recently used as tests able to simulate crewmember mission tasks (Sutterfield et al., 2019). In order to further knowledge on the physical ‘cost’ of exploratory movement in these subterranean environments, more specific studies are required. Mapping atypical movement patterns in such a subterranean environment is challenging. Accurate data collection in these challenging environments still remains predominantly a technological challenge, but it is an area where large gains could be made once movement patterns and profiling of such atypical, specialist movement are better characterized. However, even with fundamental assessment, many studies have postulated an inadequate restoration of lost calories and fluid in relation to the level and duration of movement recorded in the cave environment (Vacca et al., 1994; Bratima et al., 1999; Stenner, 2002; Stenner et al., 2007b; Antoni et al., 2017). Moreover, water losses during cave activity may also lead to acute hematological modification (i.e., increase in red blood cells and hemoglobin) as hypothesized by Stenner et al. (2007b).

Only studies in high altitude mountaineering scenarios have focused on energy expenditure over a long duration (Westerterp et al., 1992; Hoyt et al., 1994; Pulfrey and Jones, 1996; Reynolds et al., 1999; Miller et al., 2013). However, despite similarities in movement patterns, high altitude ascent does not represent a comparable model (or subsequent physiological impact) to those environmental conditions encountered in caves, especially in relation to humidity, constant temperature, and levels of hypercapnia. In order to further delineate the combined effect of multiple environmental factors found in a cave, Maura et al. (2008) tried to determine the physiological effect of an atmosphere similar to a possible cave environment at high altitude (O_2_~15%, CO_2_~1.9%, humidity 100%) during which participants completed a stress test. The given hypothesis, that when completing strenuous activity under hypoxic, hypercapnic and humid conditions, for the same given exercise an additional physiological burden and symptomology is evident and should be pre-empted in future training and analog assessments (Maura et al., 2008).

Equally, previous research related to endocrine and immune-hematological changes not only showed the potential for an anticipatory stress reaction to the cave environment, based on increased cortisol levels (Stenner et al., 2007a), but also suggested that there may be a compensatory response to both exercise and environment [evidenced by decreased cortisol levels and restoration of GH levels 24 h post cave exit (Stenner et al., 2006, 2007a,b)]. Due to the pulsatile nature of GH levels in the blood, conventional measurements of serum GH are highly variable dependent on a number of environmental stressors. GH values are usually increased with stress, exercise and low blood glucose values (Godfrey et al., 2003). Given the challenging terrain and climate intrinsic to the cave environment, caving may prove to be a strong stimulus for GH secretion. The concentration of GH in the blood can increase with time for given work intensity and can increase 10-fold during prolonged moderate exercise (Saugy et al., 2006). Therefore, long periods of potentially cyclical aerobic-anaerobic caving activity, normally almost without rest, could elicit similar increases in GH. Plus, traversing specific near vertical ascents could be considered comparable to a short duration intensive exercise (with accumulation of lactate at 70% $\overset{∙}{V}$O_2_ max for a short term period such as 10–20 min), which has previously been proven to increase GH by 5/10-fold (Felsing et al., 1992). Further long duration study of endocrinological factors, plus potential hormonal compensation, while completing exploratory tasks in the cave environment, would further add to both understanding of appetite regulation and quantification of the level of physiological stress induced during both short and prolonged active exposures. Finally, cave progression can include prolonged, multimodal, challenging traverse activity with intensive moments that can induce skeletal muscle damage and intravascular hemolysis also accompanied by the activation of inflammatory cells, as shown by the increased levels of CK and LDH, decreased level of haptoglobin and the significant increase in leucocytes due to neutrophila and monocytosis (Bregani et al., 2005; Stenner et al., 2006). These responses seem to be related both to the intensity of the exercise, pre-determined fitness/training level, overall fluid losses and the level of physiological strain induced by the specific atmospheric environmental conditions of the cave environment (Stenner et al., 2007b), and could be reduced by a specific diet. Specifically, supplementation of creatine seems to mitigate muscular damage, which typically occurs during cave progression (Bregani et al., 2005).

### Studies About Prolonged Exposure Effects in Cave Environments (Focusing on Circadian Rhythm Modifications)

During underground cave isolation, inclusive of the absence of direct interpersonal relations and external time cues, changes in the circadian system frequency are demonstrated (i.e., rest-activity cycle, rectal temperature, urine volume. and excretion of potassium) (Kleitman, 1963; Mills, 1964; Halberg et al., 1965, 1970; Reinberg et al., 1966; Siffre et al., 1966; Colin et al., 1968; Fraisse et al., 1968; Apfelbaum et al., 1969; Ghata et al., 1969; Oleron et al., 1970; Chouvet et al., 1974; Mills et al., 1974; De la Pena et al., 1989; Sonnenfeld et al., 1992; Hillman et al., 1994a,b). Consequently, a desynchronization of the biological rhythm from the environmental cycle is evident. External desynchronization is evident when a biologic rhythm becomes desynchronized from an environmental cycle. On the contrary, internal de-synchronization is described when two or more biologic rhythms in the same entity become desynchronized from each other (Halberg, 1968).

Interestingly, desynchronization with respect to local time has been observed in participants isolated in caves without time cues: in some cases individual rhythms showed external circadian de-synchronization while maintaining reasonably fixed time relations among themselves, i.e., a continuation of internal circadian synchronization (Colin et al., 1968; Mills et al., 1974; De la Pena et al., 1989; Hillman et al., 1994b; Cornelissen et al., 2010). For example, when referring to the rest-activity cycle of subject isolated in a cave for several months, a free-running circadian component was reported (i.e., independent of any rhythm in habit or environment). Nevertheless, even in the absence of natural light (or other external cues) as a synchronizer (i.e., an environmental or operative periodicity that determines the temporal placement of a given biologic rhythm by impelling the rhythm to assume synchronization), the circadian rhythm of many physiological parameters is actually maintained at the beginning of a given cave isolation. However, with a subsequent increase in the duration of cave occupation desynchronization occurs, especially related to the duration of circadian rhythm (Halberg et al., 1965). Changes were evident not only in estimated vs. actual rest-activity cycle periods and external desynchronization, but also related to body temperature, performance reaction time and heart rate cycles. Conversely, changes related to the immunological and hematological cycle, as well as the menstrual cycle and renal secretory circadian function, were reported with uncertainties.

During a forced desynchrony protocol in which light was externally controlled, Kleitman (1963) observed a persistent of a circadian rhythm of body temperature. In agreement, but under different free of time cues isolation conditions inside a laboratory, Czeisler et al. (1999) reported that the intrinsic period of the core body temperature rhythm does not appear to have been dependent on the length of the imposed sleep-wake cycle (i.e., 20 h and 28 h forced desynchronization). In fact, the temperature period estimates are nearly equivalent under both forced-desynchrony protocols (average value of 24.28 h both under 20 h and 28 h), independent of the imposed rest-activity cycle. It seems that, despite the desynchronization with respect to local time, an internal synchronization of the rhythm function was maintained due to the presence of the human circadian pacemaker.

Finally, during early cave research, study protocol usually involved the majority of participants to be hospitalized post cave isolation (Mills, 1964; Halberg et al., 1970). In doing so, they were exposed to a new influence of alternate synchronizers. Full external circadian resynchronization of all physiologic functions occurred after a return to the social routine, inclusive of the alternation of day and night, normal meal times, plus small cues such as variations in noise from hospital and urban activity (Halberg et al., 1970).

### Circadian Rhythms, Sleep and Spaceflight

Human sleep and circadian rhythms have evolved by way of adaptation to an environment characterized by a 24 h light-dark cycle and a gravitational force (Dijk et al., 2001). However, there are situations, similar to cave isolation, in which human beings are exposed to a non 24 h cycle. For example, during spaceflight astronauts are removed from the main synchronizer of the circadian system, the 24 h alternation of day-light and darkness (Monk et al., 1998). A free-run due to the absence of a 24 h light/dark cycle and increased sleep disturbances in astronauts occur more frequently during spaceflight than on the ground. Such disturbances may be caused also by exogenous factors (i.e., noise and uncomfortable temperature), but changes in the physiological basis for the regulation of sleep and the circadian clock may result specifically in sleep discomfort (Gundel et al., 1997). In fact, during spaceflight the crewmembers have reported a reduction in subjective sleep quality (Dijk et al., 2001), plus a reduction in sleep efficiency and a long sleep onset latency (Gundel et al., 1997). Decline in sleep quality may lead to a decrease in exercise performance and cognitive function (Fullagar et al., 2015), deterioration of proper immune system function (Lorton et al., 2006), and may even negatively impact upon individual probability of developing a form of cardiovascular disease (Reitz and Martino, 2015). Similarly, space flight can reportedly impact on body temperature regulation, i.e. body temperature may be increased during the imposed ”night cycle“ in comparison to normal, which may be an indication of reduced circadian amplitude (Gundel et al., 1997; Fortney et al., 1998; Polyakov et al., 2001). Moreover, spaceflight induces a pro-inflammatory response, which has also been shown to play an important role in down regulating body temperature (Luheshi et al., 1999; Crucian et al., 2014). Circadian phase, as assessed by the trough in body temperature, is delayed in astronauts during spaceflight in which they were only exposed to artificial light: the delay relative to ground-based measurements amounts to more than 2 h (Gundel et al., 1997). Spaceflight could generate circadian misalignment, which has been linked to adverse health consequences, such as impaired glucose regulation and metabolism, increased cardiovascular risk, and higher prevalence of cancers. This proposed circadian misalignment may also contribute to the use of medication (Flynn-Evans et al., 2016). One alternative countermeasure to facilitate circadian alignment is an appropriately timed exposure to both light and darkness: in fact, the circadian system is sensitive to even low levels of light (Basner et al., 2013). Specifically, the alteration of room lighting with artificial simulation of day and night may be sufficient to entrain circadian systems to a 24 h day during the preflight (Mrosovsky, 1988; Boivin et al., 1996). Aside from spaceflight, circadian disruption is also evident in terrestrial studies (Basner et al., 2013), including over-winter polar expeditions (Arendt, 2012).

Microgravity is also known to affect cardiovascular, pulmonary, neuro-vestibular, and musculoskeletal systems (Baker et al., 2008). Terrestrial partial gravity simulation models seek to simulate reduced gravity and the consequential impact it has on human physiology in relation to the proposed Lunar or Martian environment. The main problem remains that there is a lack of physiological data collected in ”real“ partial gravity scenarios and it is challenging to validate current partial gravity simulation models (Richter et al., 2017). However, in the literature to date, there are no indications that gravity directly influenced circadian rhythms and sleep in astronauts. However, in other species an effect of gravity on the circadian system is discussed (Ferraro et al., 1995). Despite this major limitation to identify space analogs suitable to directly inform extraterrestrial programs, the cave space analog has proven itself in review highly compatible to the current space environment, inclusive of EVA and isolation considerations.

### Relations and Implications for Terrestrial Space Analogs

In the cave environment there is usually a strong reduction in sunlight exposure requiring consequential adaptation to artificial light, as well as other external cues leading to augmentation of perceived vs. actual time. In addition, true isolation can be achieved, as communication can be hard to establish and prolonged confinement can be simulated under challenging conditions for days to weeks, or even months. Since 2011 ESA has organized an astronaut expeditionary training course in a space analog environment called CAVES (Cooperative Adventure for Valuing and Exercising human behavior and performance Skills) (Bessone et al., 2013; Strapazzon et al., 2014). CAVES is a scientific exploration expedition, taking place in an underground environment, to prepare international astronauts to become effective long-duration spaceflight crewmembers. Isolated cave environment allows the participants to develop autonomous operational skills in a multi-cultural team, while being challenged by an intense scientific and technological program in a surrounding that naturally recreates space-like stressors. In the 5 editions currently completed, 28 astronauts from 6 different space agencies have taken part in the training, plus more than 30 members of the scientific and organization staff stayed with them during the 7-day underground period.

A cave environment draws many parallels to current conditions on the International Space Station (ISS), inclusive the chance to replicate cues to simulate the 16 h day/night cycle, plus proposed environmental conditions for long duration interplanetary travel. Furthermore, artificial habitats will most probably be hypobaric, hyperoxic and hypercapnic in future, mainly due to the technical limitations of space travel (Norcross et al., 2013). Equally, the increase in demand for Extravehicular Activities (EVA), means increased time in the pressurized suits, which can cause thermal discomfort and are difficult to maneuver. In addition, analogous movement challenges include: the restrictive nature of cave passages, the lack of common reference points, necessity for three dimensional movement patterns, plus low visibility and perpetual darkness: all of which can reduce orientation capabilities, causing confusion and a potentially hostile perception of the environment, similar to that experienced during EVA maneuvers, or similar condition that could be found in lunar and extra-terrestrial cave (sites that could be chosen for exploration or for setting human habitat due to partial protection from cosmic radiation and stable environmental conditions compared to planetary surface). The scope of this review does not focus solely on the short effects' response, but also to prolonged effects of this atypical, subterranean environment and the potential for further development and understanding of one of the most challenging space analogs to date. This unique cave environment and the current space-agencies—led CAVES and PANGAEA (Planetary ANalogue Geological and Astrobiological Exercise for Astronauts) programs provide a unique opportunity to build upon subterranean experimental models employed previously and clearly identified here in review (Bessone et al., 2013; Strapazzon et al., 2014; Sauro et al., 2018). A more robust scientific approach, inclusive of increased numbers of trained personnel as participants could greatly improve not only the quality of research, but also the application of the results for practical considerations for future space missions. Specifically, piloting technological advances in medical monitoring equipment for real-time measurement of core temperature, cardiovascular health and energy expenditure, whilst completing specific subterranean routes and different operative conditions would help to inform estimations related to physiological strain induced under underground challenging conditions.

### Limitations

In review, structured cave research has previously lacked robust, reproducible, scientific data and is mainly limited to case studies. The reality of individual variation in response to physiological strain induced by challenging environmental conditions, even in a potentially homogeneous population such as cavers or astronauts, must be carefully considered when looking to inform practical recommendations for future space analog development. This current review identifies the need for further studies dedicated to investigating the cave environment as a space analog for exploratory missions. Precisely controlled, reproducible studies (both in the lab and cave environment) should be adopted in future, incorporating larger samples sizes, as well as a deliberate unification of protocol design, measurement validity, techniques employed, and standardized reporting. Equally, better specification and characterization of specific subterranean traverse routes and subterranean characteristics, in particular the characterization of particular obstacles, should be made to improve the level of scientific reproducibility and enable robust comparisons in terms of physiological exertion (i.e., TEE or $\overset{∙}{V}$O_2_) with alternate analogs and EVA specific tasks. A major limitation includes the pronounced methodological heterogeneity across those studies which met our selection criteria, plus the overall dearth of scientific papers which focused on this topic with sufficient clarity and thus prevented completion of a meta-analysis. It is clear that the reductive approach previously employed has led to highly fragmented understanding of current knowledge and observations gathered in the subterranean environment related to the physiological impact of a long-term exploratory cave isolation model. If these limitations were to be addressed, the cave space analog could provide the larger community an opportunity to collect novel data sets, which better inform a specific physiological strain index for subterranean activities and help delineate the physiological requirement to operate effectively in this unique environment, for the benefit of future exploratory programs.

## Conclusions

Through the systematic review, we have identified the current status of knowledge, potential gaps and areas within sports and aerospace medicine literature pertaining to the impact of subterranean exploration on human physiology that would clearly benefit from additional research efforts. The aim in the future will be to better inform operational concerns for future training and deployment in cave analogs, as well as develop specific criteria for reproducible scientific data collection related to physiological monitoring in these complex environments. Despite growing expectation from different space agencies for advancements in data collection and collation of such data, the subterranean environment has consistently proven to be one of the most challenging. Consequently, the development of a specific physiological strain index and associated protocol development for explorative caving activities and prolonged permanence is yet to be fully defined. Since previous publications focusing on caving physiology are scarce, this complex space analog calls for more specific research to better determine the physiological basis of the cave traverse, in a variety of settings and for multiple purposes.

### Future Directions

Topics highlighted within this review for further scientific investigation include: (i) *cardiovascular considerations*: cardiovascular response to both short and long duration cave exploration, with specific focus on the cardiac autonomic nervous system, heart rate variability, and potential augmentation of the sympatho-vagal balance; (ii) *environmental cost:* the metabolic impact of multiple challenging environmental factors (e.g., heat, humidity, hypoxia, hypercapnia), on subsequent estimations of TEE for a given activity and aspects of the potential performance deficit over long-duration exploratory scenarios; (iii) *basic endocrinology:* previous data on anticipatory and acute stress responses to cave exploration currently lacks translation to a prolonged model of cave isolation, mainly due to the logistical challenge of long duration isolation of large groups with relevant expertise for sufficient comparison; (iv) *hydration and hematological values:* previous work has identified potential hypo-osmolal dehydration in the case of prolonged cave traverse activity (Stenner et al., 2007b). Levels of hydration and subtle changes in plasma volume should be better determined when estimating both changes in body composition and hematological changes in muscle damage proteins and immunological markers, (v) *desynchronization:* reasonable inference exists that during specific subterranean isolation protocols there is a further requirement to closely assess self-reported rest activity cycles, renal output, menstruation and fluctuations in core body temperature, to better identify whether prolonged exposure to the cave environment, or specific ambient conditions, are responsible for further circadian desynchronization.

## Author Contributions

LB and GS developed the concept for this review. LB, GS, and LZ designed the study. Data collection and analysis were completed by LZ and LG. LZ, GS, LG, RT, EC, and LB interpreted the results. Creation of figures was completed by LZ and GS. LZ, GS, and LG wrote the article and the final draft was edited by RT, EC, and LB. All authors approved the final manuscript.

### Conflict of Interest Statement

The authors declare that the research was conducted in the absence of any commercial or financial relationships that could be construed as a potential conflict of interest.

## Abbreviations

ANS: Autonomic Nervous System
BP: Blood Pressure
BVA: Binocular Visual Acuity
CK: Serum Creatine Kinase
cpd: Cycles per degree
EE: Energy Expenditure
EEG: Electroencephalogram
EMG: Electromyography
ESA: European Space Agency
ESA CAVES: Cooperative Adventure for Valuing and Exercising human behavior and performance Skills
EVA: Extravehicular Activities
GH: Growth hormone
Hb: Hemoglobin
HCT: Hematocrit
HR: Heart Rate
HRV: Heart Rate Variability
LDH: Lactate Dehydrogenase
METs: Metabolic Equivalent of Task
NASA: National Aeronautics and Space Administration
NASA NEEMO: NASA Extreme Environment Mission Operations
PANGAEA: Planetary ANalogue Geological and Astrobiological Exercise for Astronauts
PRISMA: Preferred Reporting Items for Systematic Reviews and Meta-Analyses
PS: Paradoxical Sleep
PV: Plasma Volume
RBC: Red Blood Cell
TEE: Total Energy Expenditure.
