# Supplementary material for: Human Physiology During Exposure to the Cave Environment: A Systematic Review With Implications for Aerospace Medicine
Source: Front Physiol. 2019 Apr 24;10:442. doi: 10.3389/fphys.2019.00442 (PMC6491700; doi:10.3389/fphys.2019.00442)
Supplement: Supplementary file 1 [file Table_1.DOCX]

**Supplememtary Table 1** Additional list of specialised speleological databases consulted

| **SPECIALISED SPELOLOGICAL DATABASE** |
| --- |
| **Acta Carsologica**  (http://carsologica.zrc-sazu.si/) |
| **Australian Speleological Federation - ASF**  (http://www.caves.org.au/) |
| **Biblioteca ``F. Dal Cin`` - Grotte treviso**  ("F.Dal Cin" Library - Treviso Caves - Italy; http://bmw06.comperio.it/bmw2/speleoteca/opac.php?screen=ricerca2&loc=S&osc=ricerca2&orderby=Autore) |
| **Biblioteca ``Franco Todde`` - Federazione Speleologica Sarda**  (Sardinian speleological federation – Italy;  http://www.federazionespeleologicasarda.it/) |
| **Biblioteca ``L. Fantini`` - Gruppo Speleologico Bolognese, GSB**  (Speleological Bolognese Union, USB - Italy; http://bmw06.comperio.it/bmw2/speleoteca/opac.php?screen=ricerca2&loc=S&osc=ricerca2&orderby=Autore) |
| **Biblioteca Associazione Speleologica Bresciana**  (Brescian Speleological Association - Italy**;** http://bmw06.comperio.it/bmw2/speleoteca/opac.php?screen=ricerca2&loc=S&osc=ricerca2&orderby=Autore) |
| **Biblioteca Commissione Grotte ``E. Boegan``**  (Commisione Grotte "E. Boegan" Library - Italy; http://bmw06.comperio.it/bmw2/speleoteca/opac.php?screen=ricerca2&loc=S&osc=ricerca2&orderby=Autore |
| **Biblioteca Gruppo Puglia Grotte**  (Puglia Caves Group - Italy; http://bmw06.comperio.it/bmw2/speleoteca/opac.php?screen=ricerca2&loc=S&osc=ricerca2&orderby=Autore) |
| **British Cave Research Association**  (http://bcra.org.uk/) |
| **British Caving Association**  (http://british-caving.org.uk/wiki3/doku.php) |
| **Cave rescue Baden**  (www.hoehlenrettung-bw.de) |
| **Centre Excursionista de Catalunya**  (http://www.cec.cat/) |
| **Centro Altamurano Ricerche Speleologiche**  (Speleological Research Center of Altamurno - Italy; http://bmw06.comperio.it/bmw2/speleoteca/opac.php?screen=ricerca2&loc=S&osc=ricerca2&orderby=Autore) |
| **Centro Speleologico Romano**  (Roman Speleological Center - Italy; http://bmw06.comperio.it/bmw2/speleoteca/opac.php?screen=ricerca2&loc=S&osc=ricerca2&orderby=Autore) |
| **Chinese Mountaineering Association**  (http://bmw06.comperio.it/bmw2/speleoteca/opac.php?screen=ricerca2&loc=S&osc=ricerca2&orderby=Autore) |
| **Club Andino Bariloche**  (http://www.clubandino.org/) |
| **Croatian Cave Rescue**  (http://www.gss.hr/hgss/strucne-komisije/komisija-za-speleospasavanje/materijali-za-preuzimanje/materijali-za-preuzimanje/) |
| **European Cave Rescue Association**  (http://caverescue.eu/) |
| **Federació Catalana d'Espeleologia**  (Catalan Speleological Federation - Catalonia; http://www.espeleologia.cat/) |
| **Fédération Française de la Montagne et de l'Escalade / Commission Nationale de Secours en Montagne Français**  (French federation of mountains and climbing - France**;** http://www.ffme.fr/secours-montagne/page/commission-nationale-du-secours-en-montagne.html) |
| **Federazione Speleologica Campana, CDS ``F. Allocca``**  ("F. Allocca" Speleological Federation of Campania - Italy; http://bmw06.comperio.it/bmw2/speleoteca/opac.php?screen=ricerca2&loc=S&osc=ricerca2&orderby=Autore) |
| **Federazione Speleologica Toscana**  (Speleological Federation of Tuscany - Italy; http://bmw06.comperio.it/bmw2/speleoteca/opac.php?screen=ricerca2&loc=S&osc=ricerca2&orderby=Autore) |
| **Gruppo speleologico ```G.Chierici``**  (Speleological "G. Chierici" Group - Reggio Emilia, Italy; http://bmw06.comperio.it/bmw2/speleoteca/opac.php?screen=ricerca2&loc=S&osc=ricerca2&orderby=Autore) |
| **Gruppo Speleologico CAI Padova**  (Speleological CAI Padova Group - Italy; http://bmw06.comperio.it/bmw2/speleoteca/opac.php?screen=ricerca2&loc=S&osc=ricerca2&orderby=Autore) |
| **Gruppo Speleologico Cudinipuli**  (Speleological Cudinipuli Group - Italy; http://bmw06.comperio.it/bmw2/speleoteca/opac.php?screen=ricerca2&loc=S&osc=ricerca2&orderby=Autore) |
| **Gruppo Speleologico Natura Esplora**  (Speleological Natura Esplora Group - Summonte (AV), Italy; http://bmw06.comperio.it/bmw2/speleoteca/opac.php?screen=ricerca2&loc=S&osc=ricerca2&orderby=Autore) |
| **Hellenic Rescue Team**  (http://www.hrt.org.gr) |
| **Höhlenrettungsverbund Deutschland - HRVD**  (German Cave Rescue Assiciation - Germany;  http://hrvd.de/) |
| **Hrvatska Gorska Služba Spašavanja – HGSS**  (Croatia Mountain Rescue Service - Croatia; http://www.gss.hr/) |
| **Il soccorso alpino**  (Alpine rescue - Italy; http://www.cnsas.it/) |
| **International Climbing and Mountaineering Federation**  (http://www.theuiaa.org/) |
| **International Commission for Alpine Rescue**  (http://www.alpine-rescue.org/xCMS5/WebObjects/nexus5.woa/wa/menu?id=1063) |
| **International Journal of Speleology**  (http://scholarcommons.usf.edu/ijs/) |
| **International Society for Mountain Medicine**  (http://ismm.org/) |
| **Internet archive**  (http://web.archive.org/web/20050203090546/http://www.mauriziomontalbini.it/) |
| **Libreria Franco Anelli -**  (Franco Anelli Library**;** http://bmw06.comperio.it/bmw2/speleoteca/opac.php?BMW_Opac_Session=998c440a347b7cadeee991d663bdd845) |
| **National Speleological Society**  (http://caves.org/) |
| **Österreichischer Höhlenrettungsdienst**  (Austrian Cave Rescue Service - Austria; http://www.hoehlenrettung.at/) |
| **Parks Canada**  (http://www.pc.gc.ca/) |
| **Societé Suisse de Speleologie - SSS**  (Swiss Society of Speleology - Switzerland; http://www.speleo.ch/~site/) |
| **Speleo Secours Francaise - SSF**  (French Speleological rescue - France; http://ssf.ffspeleo.fr/fr/contacts-ssf/6-bernard-tourte) |
| **Speleolgenesis**  (http://speleogenesis.info/index.php) |
| **Wilderness Medical Society**  (http://www.wms.org/) |
