# Supplementary material for: Human Physiology During Exposure to the Cave Environment: A Systematic Review With Implications for Aerospace Medicine
Source: Front Physiol. 2019 Apr 24;10:442. doi: 10.3389/fphys.2019.00442 (PMC6491700; doi:10.3389/fphys.2019.00442)
Supplement: Supplementary file 2 [file Table_2.DOCX]

**Supplementary Table 2** List of the experts identified as candidates using citations retrieved by the search strategy (in alphabetic order)

1. Badino Giovanni - Italian Center of Speleological Documentation, Torino, Italy
2. Bakšić Ana - Speleological Section of the University Mountaineering Society "Velebit", Zegreb, Croatia
3. Bakšić Darko - Speleological Section of the University Mountaineering Society "Velebit", Zegreb, Croatia
4. Bedini Daniele - Space Architect - He worked at UNDERLAB project (lab in a cave)
5. Blancher Marc - Emergency Department - Acute Medicine, Grenoble, France (French delegate to the Medical Commission ICAR)
6. Bourges François - Geology Environment Council, Saint-Girons, France
7. Braunig Nils - German Cave Rescue Association, Germany
8. Brighetti Gianni - Psychologist, University of Bologna, Italy. He worked at UNDERLAB project
9. Budic Marko - Croatian Mountain Rescue Service, Cave Rescue Commision, Croatia
10. Bystrzinski A. - Cave Rescue Association Germany
11. Cerioni Antonella - Montalbini's wife
12. Desogus Claudia - Master thesis about case-study of a person (Sulas) isolated in a cave
13. Dodelin Christian - French Speleological rescue, France
14. Evilio Roberto - Speleological group of Faenza, Italy. He collaborated with Montalbini
15. Galvagno Andrea - Medical doctor at UNDERLAB project, Italy
16. Giovine Giuseppe - Medical doctor of La Venta association, Italy
17. Golicz Mateus - Polish Mountaineering Association, Warsaw, Poland
18. Kurt Dennstedt - Treasurer of National Association for Cave Rescue, Austria
19. Lanzoni Cristina - Experience in time-spatial isolation in cave
20. Ledoux Xavier - French National Association of Mountain Rescue Doctors, France
21. Leyk Matthias - Cave Rescue Baden Wurttemberg, Germany
22. MacGregor Ian - New Zealand Mountain Safety Council, New Zealand
23. Meyer Uli - German Caver
24. Milisic Katja - Cave - Rescue Commission Croatian Mountain Rescue Service, Croatia
25. Mocchegiani Eugenio - Immunologist, he worked for the UNDERLAB project
26. Mortimer Roger - Medical Doctor, University of California San Francisco, San Francisco, CA
27. Nordgren Marie - Swedish Mountain Rescue, Sweden
28. Novosel Dinko - Croatian mountain rescue service Speleological Section of the University Mountaineering Society "Velebit", Zegreb, Croatia
29. Paar Dalibor - Department of Physics, Faculty of Science, University of Zagreb, Croatia
30. Scarpino Osvaldo - Medical Doctor at UNDERLAB project
31. Scatolini Andrea - Speleological Journalist for Scintilena, Italy
32. Schneider Thomas - Cave Rescue Baden Wurttemberg, Germany
33. Siegenthaler Rolf - Swiss expert in caving, Burgdorf, Switzerland
34. Siffre Michel - Experience in time-spatial isolation in cave; underground explorer and scientist
35. Stenner Elisabetta - Researcher in cave physiology, University of Trieste, Department of Medicine, Surgery and Health Sciences, Italy
36. Sulas Carlo - Speleologist and experience in cave insolation
37. Tourte Bernard - French Speleology Rescue, France
38. Ubertino Alberto - National Alpine and Speleological Rescue Team, Italy
39. Westhauser Johann - German cave rescuer, Germany
40. Wolfram Gottfried - Austrian Cave Rescue, Austria
