# Supplementary material for: Human Physiology During Exposure to the Cave Environment: A Systematic Review With Implications for Aerospace Medicine
Source: Front Physiol. 2019 Apr 24;10:442. doi: 10.3389/fphys.2019.00442 (PMC6491700; doi:10.3389/fphys.2019.00442)
Supplement: Supplementary file 3 [file Table_3.DOCX]

**Supplememtary Table 3** A list of and reasons for the 19 excluded articles

| **REFERENCES** | **REASONS FOR EXCLUDE ARTICLE** |
| --- | --- |
| 700 ore sotto terra. *Novara: De agostini* In: "Franco Anelli" Library, Italy. Identification number 13164: 744-760, 1962. | No original data available |
| Antoni G, Rinaldi A, Tuveri V. Progetto di rilevamento e monitoraggio del dispendio energetico durante l'attività speleologica. Speleologia 74: 38-42, 2016. | Subsequent publication in a peer reviewed journal |
| Aschoff J. Circadian rhythms in man. *Science* 148(3676): 1427-1432, 1965. | No original data available for cave isolation |
| Bregagni ER. Disidratazione, ipotensione e shock. *Speleologia. Semestre della società speleologica italiana* 37: 91-92, 1997. | Review, no experimental data available |
| Casteret N. Les grandes heures de la spéléologie. Naissance, vie et mort des cavernes. *Librairie académique Perrin*: 303-308, 1973. | No experimental data available |
| Demaria Pesce VH. L'étude des rythmes biologiques circadiens et ultradiens de moyennes et de basses fréquences: quel intéret pour le spéléologue? *Actes des journées félix trombe* 1:58-65, 1988. | No original data about human isolated in caves |
| Guzzardi GC. L'affaticamento: fattori fisici e psichici nella pratica della Speleologia. *L'appennino* 1: 9-11, 1975. | No experimental data available |
| Mills JN. Speleology and circadian rhythms. *Transactions British Cave Research Assoc* 2(2): 95-97, 1975. | Review, no original data available |
| Mills JN. The values of speleology in the study of human rhythms. *BSA- Journal and Proceedings* 5: 49-51, 1967. | Review, no experimental data available |
| Modifiche biologiche nel compimento dello sforzo sotterraneo. Thesis In: "Franco Anelli" Library, Italy. Identification number 14925: 1-16, 1994. | Review, no experimental data available |
| Saumande P. Etude du comportement de l'homme en milieu souterrain (Bilan de cinq experimentations). *Internation Journal of speleology* 3(1): 33-41, 1968. | Weak results |
| Siffre M. Biological rhythms, sleep, and wakefulness in prolonged confinement, 1988.  [on line] https://ntrs.nasa.gov/search.jsp?R=19880016638 | No original data available |
| Siffre M. Ryhtmes biologiques, sommeil et vigilance en confinement prolonge. *ESA, Proceedings of the Colloquium on Space and Sea p 53-68(SEE N 88-26016 19-51)*, 1988. | Review, no original data available |
| Siffre M. Some problems of desynchronisation of sleep-wakefulness and circadian rhythm s for long duration spaceflights. *ESA, Proceedings of the Space e Sea Colloquium*, 1990. | No original data available |
| Siffre M. Sur quelques aspects de psyche-physiologie humaine en speleologie. *Como: Rassegna Speleologica Italiana*. In: "Franco Anelli" Library, Italy. Identification number 21933 :1-8, 1963. | Personal experience in underground isolation, no experimental data available |
| Stenner, E. Adattamenti biochimici alla speleologia alpina. *EUT Edizioni Università di Trieste*, 2013. | No original data available |
| Taylor HL. A physiological effect of cave visiting. *Science* 12; 21(536): 263, 1893 | No data available |
| Vacca U, Tuveri V, Irsara S. Monitoraggio della funzione cardiovascolare in speleologia. *Grotte, attività e riflessioni della commissione E. Boegan* *"PROGRESSIONE 30",* 1994. [online] http://www.boegan.it/1993/01/relazione-medica-tepuy-93/ | No experimental data available |
| Veljkovich M. Foundation for mind research in extended sensory deprivation. *Newsletter* 1: 1-3, 1995. | Personal experience in underground isolation but no experimental data available |
